# A Review of the In Vivo Evidence Investigating the Role of Nitrite Exposure from Processed Meat Consumption in the Development of Colorectal Cancer

**DOI:** 10.3390/nu11112673

**Published:** 2019-11-05

**Authors:** William Crowe, Christopher T. Elliott, Brian D. Green

**Affiliations:** Institute of Global Food Security, School of Biological Sciences, Queens University Belfast, Belfast BT9 5DL, UK; w.crowe@qub.ac.uk (W.C.); chris.elliott@qub.ac.uk (C.T.E.)

**Keywords:** nitrite, processed meat, colorectal cancer

## Abstract

The World Cancer Research Fund (WCRF) 2007 stated that the consumption of processed meat is a convincing cause of colorectal cancer (CRC), and therefore, the public should avoid it entirely. Sodium nitrite has emerged as a putative candidate responsible for the CRC-inducing effects of processed meats. Sodium nitrite is purported to prevent the growth of *Clostridium botulinum* and other food-spoiling bacteria, but recent, contradictory peer-reviewed evidence has emerged, leading to media reports questioning the necessity of nitrite addition. To date, eleven preclinical studies have investigated the effect of consuming nitrite/nitrite-containing meat on the development of CRC, but the results do not provide an overall consensus. A sizable number of human clinical studies have investigated the relationship between processed meat consumption and CRC risk with widely varying results. The unique approach of the present literature review was to include analysis that limited the human studies to those involving only nitrite-containing meat. The majority of these studies reported that nitrite-containing processed meat was associated with increased CRC risk. Nitrite consumption can lead to the formation of N-nitroso compounds (NOC), some of which are carcinogenic. Therefore, this focused perspective based on the current body of evidence links the consumption of meat containing nitrites and CRC risk.

## 1. Introduction

It has been reported that between 66% and 99% of Europeans consume processed meat, with the mean amount consumed per day ranging between 10 and 80 g per day [1]. Processed meats are defined as meats that have been modified through curing, fermentation, salting, smoking, or otherwise, for the purpose of improving shelf life and/or enhancing flavour. Red meat is a nutritionally important source of protein, providing all essential amino acids and minerals, such as iron, selenium, and zinc. In 2007, the World Cancer Research Fund (WCRF) stated that there is convincing evidence linking the consumption of red and processed meat with the development of colorectal cancer (CRC) [2]. The WCRF further stated that the public should limit their intake of red meat to below 500 g per week, and avoid processed meat entirely. A further update from the WCRF emphasized that no safe level of processed meat could confidently be attributed to a lack of risk [3]. The International Agency for Research on Cancer (IARC) stated that with every increase of 50 g of processed meat consumption per day, the risk of CRC rises by 18%, whilst with every increase of 100 g of red meat consumed per day the risk of CRC rises by 17% [4]. Numerous meta-analyses have been conducted in this area, the majority of which have reported processed meat to be linked to CRC development [5,6,7,8,9,10,11,12]. Due to the narrow scope of a meta-analysis, no animal evidence is included in the synthesis, but animal studies are well controlled, so it is important to consider them in the balance of evidence.

Genetic risk factors are undoubtedly involved in CRC development; however, increasing evidence suggests a more minor role than previously thought. Studies mapping the geographical incidences of cancer have noted that incidences in immigrants begin to reflect that of the host nations’ incidences within a single generation [13], indicating that environmental factors are the largest contributor to CRC development.

A number of components present in processed meat have been implicated as potential causes of CRC including heterocyclic amines (HCA), polycyclic hydrocarbons (PAH), nitrites, haem iron, and high fat. HCA and PAH are also present in other foodstuffs that are not associated with CRC, such as fish and poultry, and therefore, have been largely ruled out [14]. Nitrites have emerged as a leading candidate responsible for processed meats’ association with CRC; however, it is possible that a combination of the constituents listed could coalesce to initiate the pathogenesis of CRC. Nitrites are an effective preservative that uniquely prevent the growth of *clostridium botulinum*. Nitrites also enhance the colour and flavour of processed meat. Nitrite consumption can lead to the endogenous formation of N-nitroso compounds (NOC), some of which are carcinogenic [15].

The WCRF has extensively reviewed the existing research concerning the effect of processed meat consumption and CRC and their conclusions appear to be definitive. There is good reason to believe that a causal relationship exists; however, there is not consensus of opinion in the scientific literature. This review analyses the conflicting preclinical and clinical research investigating the role of processed meat consumption in the development of CRC. There is a specific focus on the relationship between nitrite-containing processed meat and CRC.

### 1.1. Methods

**Figure 1:**
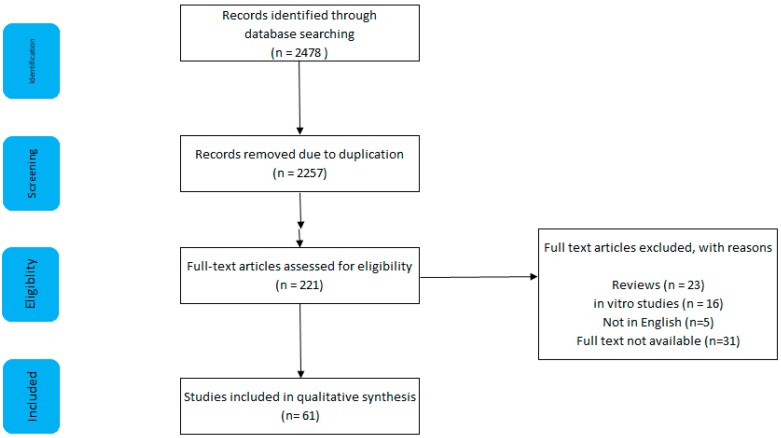
Search strategy and results, including reasons for exclusion.

As shown in Figure 1, a literature search was conducted using 3 search engines: Pubmed, Scopus, and Web of Science. A combination of the following terms were searched: colorectal cancer, CRC, processed meat, sausage, bacon, nitrite, and *N*-nitroso compounds.

### 1.2. Inclusion and Exclusion

Studies were included provided that they met the following criteria: full text was available, written in English, including single or multiple measures of colorectal cancer; that it was a human study that included a measurement of processed meat consumption. Reviews and technical reports were excluded. Studies that only utilised in vitro techniques were excluded.

### 1.3. Results

The search yielded 2478 results, of which 61 were deemed to be appropriate. A total of 2257 articles were excluded owing to duplication; five articles were not in English; full text was not available for 31 articles; 23 reviews/technical reports/letters to editors were excluded; 16 studies used only in vitro techniques; 85 human studies did not measure processed meat consumption.

## 2. Preclinical Evidence

As shown in Table 1, eleven preclinical studies have investigated the effect of nitrite-containing processed meat consumption on CRC development. Eight studies employed Fischer rats, one employed Sprague Dawley rats, one employed Apc^Min^ mice, and one used A/J mice and CF-1 mice.

Unfortunately, the Apc^Min^ study exposed animals to nitrite supplemented water, and therefore, did not supplement processed meat with nitrite. In nine of the studies, the development of CRC was confirmed by the occurrence of aberrant crypt foci (ACF) or mucin depleted foci (MDF). One study measured faecal levels of NOC and one measured total N-nitroso compounds, cytotoxicity, and thiobarbituric-acid-reactive substances.

Three of the eleven studies concluded that nitrite exposure did not increase the risk of CRC [16,17,18]. One found that hot dog did not increase the risk but 1.5 g sodium nitrite/L did [19]. Five studies concluded that nitrite exposure resulted in an increased risk of CRC [20,21,22,23,24]. Mirvish et al. 2003 reported that nitrite consumption increased NOC excretion but did not measure any CRC outcomes [25]. One study reported that nitrite ingestion elicited a protective effect on CRC development [26]. Those studies which found that nitrite-containing processed meat was causative for CRC, all exposed animals to a dietary amount of at least 50% processed meat. Although potentially achievable in the human diet, it seems unrealistic. Conversely, the only study which found processed meat to be protective for CRC provided a 60% meat diet for a period of 100 days. One explanation for the different outcome of this study is that the processed meat (bacon) group contained much higher NaCl levels than the control. This increases water intake and subsequently raises faecal moisture content. The intestinal dilution of nitrous compounds is one possibility for the reported findings.

Two studies that reported a causative relationship did not have adequate control groups to conclude that the findings were due to nitrite [22,23]. Furthermore, neither had a negative control, and were both are unable to conclude that processed meat increases the risk of CRC compared with a meat-free diet. Two studies reporting no effect used nitrite-containing water rather than nitrite in a food matrix [16,23]. In these studies, nitrite and haem iron were provided concomitantly, but not protein. The carcinogenic NOCs form most efficiently in the presence of high levels of nitrite, iron, and protein [27]. Therefore, studies which add nitrite alone or in the absence of either iron and protein, will not realise the full carcinogenic effect. The above two studies were also the only studies not to actually measure ACF and MDF.

The majority of studies investigating nitrite causation of CRC measured ACF and MDF as the primary outcomes. Adenomas typically take several months to manifest, whilst ACF and MDF develop in a matter of weeks. Therefore, ACF and MDF are used widely used, especially as there is a high concordance between the quantity of ACF and MDF and the development of adenomas. ACF is an early histopathological indicator of CRC, but it should be pointed out that not all ACF lesions lead to clinical CRC. These are easily identifiable pre-neoplastic lesions that are typically present on the mucosal surface. MDF are a subset of ACF, the primary difference being that mucous production is suppressed in MDF. Visual inspection of the colon remains the gold standard for confirming the determination of disease pathology. Although there are putative blood, urinary, and faecal biomarkers, none of them are as specific and sensitive as ACF and MDF [28,29].

In summary, the existing preclinical research investigating the effect of nitrite exposure on CRC development is conflicting and methodological inconsistencies are apparent. Furthermore, the lack of a dose-response study is a major deficiency. The heterogeneous nature of chemical-induced cancer in animal models can be problematic, due to the unpredictability of the location and timing of cancer development [30]. Despite the variability that ensues, the majority of existing studies have used chemically induced models. Future studies should investigate models spontaneously developing CRC, such as the Apc^Min^ mouse. This is a murine model that is susceptible to colorectal adenomas, in which there is a deletion of the APC gene. It is similar to the human hereditary condition, familial adenomatous polyposis (FAP). The use of in vivo models with similar pathogeneses to human conditions will increase the ability to extrapolate the findings.

Differences in physiological processes exist between humans and murine models, preventing direct extrapolation of findings. For example, the microflora of humans and rats differ and given that bacteria can increase the formation of nitrosamines [31]. Such differences may have a considerable effect on the association between nitrite consumption and CRC development. It is, therefore, imperative to give greater credence to human studies.

## 3. Clinical Evidence

A large number of studies of differing designs have investigated the relationship between human processed meat consumption and CRC. Table 2; Table 3, respectively, describe all prospective studies and case-control studies conducted thus far. Supplementary Tables S1 and S2 include more information about the studies presented in Table 2 and Table 3. Of the 49 human studies identified, 23 found that processed meat consumption was linked with CRC [32,33,34,35,36,37,38,39,40,41,42,43,44,45,46,47,48,49,50,51,52,53,54], 25 found no link [55,56,57,58,59,60,61,62,63,64,65,66,67,68,69,70,71,72,73,74,75,76,77,78,79], and one study found processed meat to be protective for CRC [80]. It is, therefore, difficult to draw any definitive conclusions from these studies.

Although an equal number of studies prove/disprove the link between processed meat and CRC, those studies which found a relationship generally involved more participants; this explains why numerous meta-analyses have concluded that processed meat is a risk factor for CRC [5,6,7]. One study reported no association between processed meat consumption and CRC in men (*n* = 241), but reported a beneficial association between processed meat and CRC in women (*n* = 197) [69]. Two further prospective studies found that processed meat consumption was associated with CRC in men but not in women [37,59]. These findings were supported by three further studies (*n* = 119,260) indicating no effect of processed meat in female cases and controls [32,55,79]. A study of almost 48,000 American men reported that the association between processed meat consumption and CRC did not reach significance (*p* = 0.06) [73].

### Studies Focusing on Nitrite-Containing Meat and CRC

Of the human studies discussed above, 17 studies investigated nitrite-containing processed meats (Table 4). Five studies found nitrite-containing processed meats to have no effect on CRC [43,56,57,59,62] and one study found nitrite-containing processed meat was protective [80]. In contrast, a total of 11 studies found that nitrite-containing processed meat increases the risk of CRC [34,35,36,37,38,39,42,45,48,49,53]. This indicates that the proposed causal relationship between processed meat is potentially skewed by the intake of nitrite-containing processed meats.

Of the prospective studies discussed (*n* = 5), three used the International Classification of Diseases criteria to confirm that the participant met the conditions of a positive diagnosis of CRC [49,57,59]; two studies did not specify which criteria they used. Wu et al. [48] consulted the patients’ medical records. Takachi et al. [56] conducted a linkage study, where the researchers had access to a local cancer registry. Of the case-control studies (*n* = 12), ten studies recruited participants that had histologically confirmed adenocarcinoma; only one stated that the International Classification of Diseases criteria was applied to their study population [80]. Dales et al. [62] recruited hospitalized CRC patients, whilst Young and Wolf [37] recruited participants from the Wisconsin Cancer Reporting System.

All studies used a food frequency questionnaire (FFQ) to capture processed meat consumption in their respective cohorts; only two of the studies set out with the intention of investigating the role of nitrite in CRC development [59,62] and both of these found no relationship. No study considered the concomitant consumption of haem proteins, and only one study considered the effect of nitrosamine exposure [59] levels; however, no study directly measured that. The FFQs varied in design and detail. Three studies recorded information on portion size [43,45,57], and the remainder did not. Researchers invited participants to describe their consumption habits in a number of ways, Dales et al. [62] included eight different frequencies, ranging from “never” to “at least once a day.” Four studies provided their participants with six frequencies to choose from [34,37,48,80]. Two studies used five categories of frequency [42,57], Lohsoonthorn et al. [53] used four categories; the remainder of the studies did not specify how many categories were available (*n* = 10). Only one study described their FFQ as validated [39]. It has been shown that illness substantially effects dietary intake, and it is, therefore, crucial that all studies investigating the causative effect of habitual dietary pattern, consider the period prior to illness. Of the studies that recruited participants with existing CRC (*n* = 12), seven studies stated that participants were instructed to record dietary information on periods prior to illness. These periods ranged from weekly to over the course of the participant’s lifetime.

The above analysis of prior studies clearly indicates that there is a need for methodical studies which specifically investigate nitrite exposure, and which control for confounding factors, such as haem, and saturated fat intake. As a pre-requisite, a well-designed, validated FFQ focusing on processed meat must include portion size. An early report concluded that although processed meat consumption was linked to CRC development [36], nitrite was not responsible. This conclusion was based on the finding that meat with no nitrite had a higher relative risk than meat with nitrite [36]. The authors postulated that meat consumption was acting as a surrogate measure of saturated fat consumption, and that in fact, saturated fat intake could be responsible for the positive association [36]. Further investigation found that the frequency of consumption of nitrite-containing foods and high fat containing foods were not different between cases or controls [62]. This hypothesis is disputed by a study that found controlling for meat intake substantially decreased the association between fat intake and CRC development, suggesting that meat intake was responsible for the relationship [81]. 

Positive relationships between CRC and the consumption of salami [38], sausages [39], ham [34], and bacon [45] have been reported, although Sato et al. [57] found no relationship between CRC and sausage or ham intake. The content of a sausage differs greatly depending on the location it is being manufactured in. Sausages made in continental Europe tend to contain sodium nitrite, whilst British/Irish sausages do not. As the aforementioned studies that measured sausage intake were conducted in Argentina and Japan, respectively, it is difficult to determine the proportion of nitrite-containing sausages that the populations actually consumed.

In a prospective study of 9985 participants, it was reported that *N*-nitrosodimethylamine consumption was positively related to CRC development; when locating the origin of the *N*-nitrosodimethylamine, there was a strong significant association between intake of smoked and salted fish and risk of CRC; however, the association between intake of cured meat and sausages with CRC was not significant [59]. A case-control study found that CRC patients were more likely to consume processed lunchmeat at various stages of their life compared with controls, and furthermore, it was speculated that additives such as nitrites may be responsible for this association [37]. Pierre et al. (2013) [22] conducted the only human intervention study in this area. Seventeen males were asked to abstain from meat and antioxidants for a 7-day control period, following which they consumed 180 g of ham per day for 4 days; after a washout period, the same participants consumed 180 g of tocopherol enriched ham a day for 4 days, and finally, 180 g of ham and 500 mg of calcium per day. During the ham-only period, participants had significantly higher faecal levels of apparent total N-nitroso compounds (ATNC) and thiobarbituric acid reactive substances (TBARS) than during the control periods. The addition of calcium to the diet mitigated this rise. There was no change in the faecal water cytotoxicity or 1,4-Dihydroxynonane mercapturic acid (DHN-MA) following any treatment. None of the markers measured in that study are validated markers of colorectal cancer; however, they are the best available without imaging or visualising the colon. The existing human evidence indicates that nitrites, through their exogenous and endogenous conversion to NOCs, are an important contributing factor in the proposed causal link between processed meat consumption and CRC.

## 4. Potential Mechanisms

Up to 75% of NOC exposure can be attributed to endogenous production [82]. NOCs are formed following the ingestion of nitrite. The mechanism responsible for this is the electron exchange reaction involving amines and nitrogen oxides; this occurs most efficiently when at low pH [83]. NOCs are also encountered from exogenous sources, such as cigarette smoking and the diet. Nitrosamines are also exogenously produced, commonly occurring in foods such as cheese, fish, beer, and water. Common forms of exogenous derived nitrosamines are *N*-nitrosodimethyamine (NDMA); *N*-nitrosodiethylamine (NDEA); Nnitrosodibutylamine (NDBA); and *N*-nitrosopiperidine (NPIP). Not all of these compounds are carcinogens but some have been classified as probable by IARC [84].

The enzymatic incorporation of a hydroxyl group to *N*-nitrosamines catalysed by cytochrome P450 2E1 (CYP2E1) yields diazomethane, and ultimately, DNA-reactive methyl carbocation [85]. This involves the formation of methylated DNA adducts, by the addition of an alkyl group to oxygen within DNA bases, which leads to the production of two mutagenic nucleobases, *O*^6^–methylguanine and *O*^4^–methylthymine [86]. The relationship between CYP2E1 and NOC metabolism is so profound that it has been reported that depletion of CYP2E1 confers resistance to smoking-induced lung cancer [87]. Haem centres can reduce nitrite to nitric oxide (NO), and depending on substrate availability, nitrosation can then occur. It has been shown that the nitrosation of amines can lead to the production of diazoacetate and ultimately *O*^6^-carboxymethyl-2′-deoxy-guanosine, which is a further NOC DNA adduct [88]. 

Multiple nutrients have been shown to prevent the formation of NOCs, including vitamin C, vitamin E, and chlorophyll. It is well established that vegetables contain significant amounts of nitrates which can be reduced by bacteria during mastication to form nitrite, and this nitrite enters the stomach for nitrosation. However, as vegetables do not contain haem, instead containing antioxidants such as vitamin C, vitamin E, and chlorophyll, the nitrite derived from vegetables does not pose the same mutagenic risk as processed meat. 

## 5. Discussion

CRC is a somatic genetic disease and its onset is influenced by both the colonic environment and the patient’s genetic background. Increased CRC risk is evident in populations of particular dietary patterns. Studies that have focused on singular foodstuffs or nutrients have frequently yielded inconclusive findings, indicating that the risk of CRC is altered depending on dietary pattern rather than simply the consumption of a particular food. Thus, by far the majority of studies have not considered the cumulative effects of multiple risk factors, including increased abdominal adipose tissue, cigarette smoking, physical inactivity, alcohol intake, consumption of saturated fats, and low fibre consumption. The long-term, habitual consumption of processed meat is likely to be another contributing factor [89,90]. It is evident from the pre-clinical studies that haem is a promotor of CRC development; however, it is unclear from the human evidence if it is simply a confounding factor or an important contributor. The majority of human studies conducted have included multivariate analysis to control for confounding factors; however, too frequently the researchers rely on self-reported data. It is clear from the proposed mechanism that nitrite requires other nutrients to be present to produce carcinogenic compounds. Future research should take a holistic dietary approach when analysing exposure data. 

The majority of nitrates ingested in the diet come from vegetable consumption. As previously stated, nitrates are reduced during mastication to form nitrite; the acidic environment of the stomach encourages further reduction to create NO and S-nitrosothiols. In conflict with the conclusions of this review, these by-products of nitrites can elicit beneficial health effects; acidified nitrite can enhance the stomach’s antimicrobial activity. Specifically, acidified nitrite has been shown to prevent the growth of *Salmonella enteriridis*, *Salmonella typhumurium*, *Yersinia enterocolitica*, *Shigella sonnei*, and *Escherichia coli* (DYK) [91]. NO has also been shown to be a potent vasodilator, and S-nitrosothiols are known to modulate platelet function. These two functions coalesce to improve cardiovascular function, through a lowering of blood pressure. Oxides of nitrogen react with unsaturated fatty acids, such as conjugated linoleic acid, to form nitroalkenes, and these have been shown to inhibit proinflammatory cytokines, such as endothelial TNF-α [92]. 

The association between processed meat and CRC is more pronounced in those studies considering only nitrite-containing processed meats. However, the relationship still remains modest in comparison to established carcinogens, such as smoking, that exhibit relative risks in excess of 100 fold [93]. Many of the human studies supporting a role for processed meat in colorectal cancer pathogenesis suffer from methodological limitations. Conversely, the preclinical studies are well controlled, yet yield conflicting results. Preclinical studies indicate that that low processed-meat-consumption does not increase CRC risk; however, high consumption does. It is reported that NOC levels are higher in the faeces of rodents consuming nitrite than in controls [17]; however, this increase does not result in an increased incidence of adenomas, and that is because not all NOCs are carcinogenic [94]. An unintended consequence of the addition of sodium nitrite to meat is the interaction with secondary amines and the formation of NOCs; a wide range of NOCs exist and the individual risk of each is yet to be characterised. It is possible that the discordance in results is explained by a diverse range of NOCs being formed in each preclinical study.

The use of nitrites as curing agents is controversial. The findings summarised in this review raise more concerns with its usage. It has been reported that the removal of nitrite from processed meat does not compromise its safety [95]; this issue has attracted considerable media attention [96]. It is clear that well-designed, peer-reviewed research that investigates the efficacy of sodium nitrite as a preservative is needed.

Future epidemiological studies should consider more specifically categorising the processed meat consumed, explicitly distinguishing between those meats which are nitrite-containing and those which are not. When this type of data becomes available it will be possible to infer the actual risk posed by the addition of nitrites. Furthermore, preclinical dose response studies are currently lacking and these could help refine the no observed adverse effect level (NOAEL), informing the design of human intervention studies. On the basis of several observational studies [32,34,57,74], the WHO has declared that processed meat should be avoided entirely. Currently, all processed meats are considered group 1 carcinogens, despite there being a vast difference in nutrient compositions. Some processed meats do not contain nitrite; therefore, it is possible that not all processed meats carry the same level of risk.

## Figures and Tables

**Table 1:** Characteristics of animal studies assessing colorectal cancer and nitrite consumption

Author	Model	Intervention	Fat Content	Control	Outcome
[17] Parnaud et al. 1998	Fischer rat	30% bacon (freeze dried), 70% AIN-76 for 100 days 60% bacon for 30 days	7%, 14%, 28%	AIN-76 formula with identical protein and fat (Casein and lard used to increase macros)	↑ Fecal NOC level No ACF were detected in the colon of bacon-fed uninitiated rats.
[26] Parnaud et al. 2000	Fischer rat	30% bacon (freeze dried), 70% AIN-76 for 100 days 60% bacon for 100 days	14% 28%	AIN 76 formula with identical protein and fat (casein, olive oil and lard used to increase macros)	↓ACF by 12% in rats fed a diet with 30% bacon and by 20% in rats fed a diet with 60% bacon.
[25] Mirvish et al. 2003	Sprague– Dawley rats and male mice of various strains	18% hot dog, 82% TD-01407 SP 7 days 15% beef. 82% TD-01407 SP 7 days	26 g/100 g	TD-01407 TD-98061 AIN-76A diet soy oil and casein added to increase macros	↑ Fecal NOC in hot dog and beef fed compared with control.
[20] Santarelli et al. 2010	Fischer rat	55% processed pork (moist), 45% AIN76	15 g/100 g	AIN-76A	↑ MDF in processed pork group.
[21] Pierre et al. 2010	Fischer 344 rats	55% cured ham (freeze dried), 45% AIN76A	-	Ain-76A	↑ ACF and MDF in cured ham fed group.
[21] Santarelli et al. 2013	Fischer rat	55% processed meat (moist), 40% AIN76, 5% safflower oil	30%	AIN-76A with 5% safflower	↑ MDF in hot dog fed group. Addition of calcium carbonate suppresses lesions.
[16] Chenni et al. 2013	F344 rats	AIN-76 with sodium nitrite in drinking water (1 g/L) nitrite (0.17 g/L) and nitrate (0.23 g/L)	-	AIN-76A	No change.
[22] Pierre et al. 2013	Fischer 344 rats	55 g (moist weight) experimental cured meat, 45 g AIN76 100 days	15%	AIN-76A	↑ MDF in cured meat fed group, compared with vitamin E and calcium supplemented groups.
[18] Bastide et al. 2015	F344 ratsC57BL/6J ApcMin/+ miceApc+/+ mice	(0.17 g/L of NaNO_2_ and 0.23 g/L of NaNO_3_) added to water AIN-76A with 2.5% haemoglobin	-	AIN-76A	↑ MDF in heme iron fed group. No change in nitrite fed group.
[19] Zhou et al. 2015	A/J mice CF-1 mice	0.5 or 1.0 g NaNO_2_/L 0, 1.0, 1.25, or 1.5 g NaNO_2_/L 18% hot dog	-	AIN93G	No change following hot dog ingestion. ↑ ACF in 1.5 g compared with untreated
[23] Bastide et al. 2017	Fischer 344 rats	50 g cooked, cured meat, 50 g AIN76 100 days vs polyphenol rich diet	-	Ain-76A	↑ MDF in cured meat fed group.

↑ increased; ↓ decreased; ACF Aberrant crypt foci; CRC colorectal cancer; NOC N-nitroso compound level; MDF mucin depleted foci.

**Table 2:** Characteristics of prospective human studies assessing colorectal cancer and processed meat consumption

Author	Sample Size	Colorectal Cancer Cases	Description of Processed Meat	Relative Risk (CI)	Findings
[32] Oba et al. 2005	31,552	213	Processed meat	1.98 (1.24–3.16)♂ 0.85 (0.50–1.43)♀	Significantly ↑ risk of CRC in ♂ not ♀
[33] Goldbohm et al. 1994	3123	393	Processed meat	1.72 (1.03–2.87)	Significantly ↑ risk of CRC
[44] Parr et al. 2013	84,210	674	Processed meat	1.54 (1.08–2.19)	Significantly ↑ risk of CRC
[48] Wu et al. 2006	14,032	581 ‡	Processed meat	1.52 (1.12–2.08)	Significantly ↑ risk of CRC
[55] Bostick et al. 1994	35,215	212	Processed meat	1.51 (0.72–3.17)	No significant risk of CRC
[49] English et al. 2004	37,112	451	Processed meat	1.50 (1.1–2.0)	Significantly ↑ risk of CRC
[50] Norat et al. 2005	478,040	1329	Processed meat	1.42 (1.09–1.86)	Significantly ↑ risk of CRC
[56] Takachi et al. 2011	98,514	1145	Processed meat	1.27 (0.95–1.71)♂ 1.19 (0.82–1.74)♀	No significant risk of CRC
[51] Willet et al. 1990	88,751	150	Processed meat	1.21 (0.53–2.72)	Significantly ↑ risk of CRC in the 3rd quintile but not the 4th
[67] Pietinen et al. 1999	27, 111	185	Processed meat	1.20 (0.7–1.8)	No significant risk of CRC
[52] Cross et al. 2007	494,036	5107	Processed meat	1.20 (1.09–1.32)	Significantly ↑ risk of CRC
[46] Bradbury et al. 2019	468,910	2576	Processed meat	1.19 (1.01–1.41)	Significantly ↑ risk of CRC
[73] Giovannucci et al. 1994	47,949	205	Processed meat	1.16 (0.44–3.04)	No significant risk of CRC
[47] Cross et al. 2010	300,948	2719	Processed meat	1.16 (1.01–1.32)	Significantly ↑ risk of CRC
[74] Chao et al. 2005	148,610	1197	Processed meat	1.13 (0.91–1.41)	No significant risk of CRC
[75] Lee et al. 2009	74,942	394	Salted meat	1.10 (0.8–1.4)	No significant risk of CRC
[76] Larsson et al. 2005	61,433	234ⱡ 155‡	Processed meat	1.07 (0.85–1.33)	No significant risk of CRC
[77] Ollberding et al. 2012	215,000	3404	Processed meat	1.06 (0.94–1.19)	No significant risk of CRC
[78] Egeberg et al. 2013	53,988	914	Processed meat	1.02 (0.78–1.34)	No significant risk of CRC
[79] Flood et al. 2002	45,496	487	Processed meat	0.97 (0.73–1.28)	No significant risk of CRC
[57] Sato et al. 2006	47,605	358	Ham or sausage	0.91 (0.61–1.35)	No significant risk of CRC
[58] Lin et al. 2004	37,547	202	Processed meat	0.85 (0.53–1.35)	No significant risk of CRC
[59] Knekt et al. 1999	9985	73	Nitrite	0.74 (0.34–1.63)	No significant risk of CRC

♂ male; ♀ female ↑ increased; ⱡ Proximal colon; ‡ distal colon; CRC colorectal cancer. Where the authors did not provide the relative risks for males and females combined, we have provided the gender specific relative risks. Relative risk values reflect those in the highest consumption group vs those in the lowest consumption group. All relative risk values are adjusted, more details are provided in Supplementary Table S1.

**Table 3:** Characteristics of case control human studies assessing colorectal cancer and processed meat consumption

Author	Cases (*n* =)	Controls (*n* =)	Description of Processed Meat	Relative Risk (CI)	Findings
[53] Lohsoonthorn et al. 1995	279	279	Bacon	12.49 (1.68–269.1)	Significantly ↑ bacon consumption in cases group
[54] De Stefani et al. 2012	321	844	Processed meat	3.53 (1.93–6.46) 2.01 (1.07–3.76)	Significantly ↑ risk of CRC
[34] Tajima and Tomina 1985	93	186	Ham and sausage	2.87	Significantly ↑ risk of CRC
[35] Levi et al. 2004	323	1271	Processed meat	2.53 (1.50–4.27)	Significantly ↑ risk of CRC
[36] Haenszel et al. 1973	179	357	Sausage and other processed pork	2.30Ϟ 1.77Ф 2.7҂	Significantly ↑ risk of CRC
[37] Young and Wolf 1988	152 ⱡ 201 ‡	618	Processed lunch meat	1.85 (1.33–2.58)	Significantly ↑ risk of CRC
[38] Bidoli et al. 1992	123¥ 125₸	699	Salami and sausages	1.8¥ 1.9₸	Sig ↑ ¥ but not ₸
[39] Navarro et al. 2003	287	566	Cold cuts and sausages	1.64 (1.16–2.32)	Significantly ↑ risk of CRC
[40] Rosato et al. 2013	329	1361	Processed meat	1.56 (1.11–2.20)	Significantly ↑ risk of CRC
[41] Hu et al. 2008	3174	5039	Processed meats	1.50 (1.2–1.8)	Significantly ↑ risk of CRC
[60] Williams et al. 2010	945	959	Processed meat	1.36 (0.80–1.68)Ւ 1.02 (0.38–1.96)ⱦ	No significant risk of CRC
[61] Benito et al. 1990	286	498	Processed meat	1.36	No significant risk of CRC
[42] De Verdier et al. 1991	559	505	Sausage	1.30 (0.8–1.9)¥ 1.7 (1.1–2.8)₸	Significantly ↑ risk of CRC
[62] Dales et al. 1978	99	280	Nitrite treated meats	1.22	No significant risk of CRC
[63] Joshi et al. 2015	3350	3504	Processed meats	1.20 (1.0–1.4)	No significant risk of CRC
[64] Balder et al. 2006	1535	4371	Processed meats	1.18 (0.84–1.64)	No significant risk of CRC
[65] Murtaugh et al. 2003	952	1205	Processed meat	1.18 (0.87–1.61) ♀ 1.23 (0.84–1.81) ♂	No significant risk of CRC
[66] Kimura et al. 2007	782	793	Processed meats	1.15 (0.83–1.60)	No significant risk of CRC
[68] Nothlings et al. 2009	1009	1522	Processed meat	1.08 (0.89–1.39)	No significant risk of CRC
[69] Steinmetz and Potter 1993	220	438	Processed meat	1.03 (0.55–1.95)♂ 0.77 (0.35–1.68)♀	No significant risk of CRC
[70] Franceschi et al. 1997	1225	4154	Processed meat	1.02 (0.89–1.24)	No significant risk of CRC
[71] Centozone 2009	119	119	Processed meat	1.01	No significant risk of CRC
[72] Tiemersma et al. 2002	102	537	Sausage	0.90 (0.6–1.3)	No significant risk of CRC
[43] Macquart-Moulin 1986	399	399	Charcuterie	0.89	Significantly ↑ risk of CRC
[80] Iscovich et al. *1992*	110	220	Processed meat	0.43 (0.21–0.89)	Significantly ↓ risk of CRC
[45] Nowell et al. 2002	157	380	Sausage and bacon	-	Significantly ↑ bacon consumption in cases group

↑ increased; ↓ decreased ⱡ Proximal colon; ‡ distal colon; ♂ male; ♀ female; ¥ colon; ₸ rectal; Ւ caucasion; ⱦ African American; CRC colorectal cancer. Where the authors did not provide the relative risks for males and females combined, we have provided the gender specific relative risks. Relative risk values reflect those in the highest consumption group vs those in the lowest consumption group. All relative risk values are adjusted, with the exception of Iscovich et al. and Nowell et al. more details of adjustments are provided in Supplementary Table S2.

**Table 4:** Characteristics of prospective and case control human studies assessing colorectal cancer and nitrite containing meat

**Author**	**Sample size**	**Colorectal Cancer Cases**	**Description of Processed Meat**	**Relative Risk (CI)**	**Findings**
[48] Wu et al. 2006	14,032	581 ‡	Sausage, salami, bologna	1.52 (1.12–2.08)	Significantly ↑ risk of CRC
[49] English et al. 2004	37,112	284	Salami, continental sausages, sausages or frankfurters, bacon, ham	1.50 (1.1–2.0)	Significantly ↑ risk of CRC
[56] Takachi et al. 2011	98,514	1145	Processed meat	1.27 (0.95,1.71) ♂ 1.19 (0.82,1.74) ♀	No significant risk of CRC
[57] Sato et al. 2006	47,605	358	Ham or sausage	0.91 (0.61–1.35)	No significant risk of CRC
[59] Knekt et al. 1999	9985	73	Nitrite	0.74 (0.34–1.63)	No significant risk of CRC
**Author**	**Cases (*n* =)**	**Controls (*n* =)**	**Description of Processed Meat**	**Relative Risk (CI)**	**Findings**
[53] Lohsoonthorn et al. 1995	279	279	Bacon	12.49 (1.68–269.1)	Significantly ↑ bacon consumption in cases group
[34] Tajima and Tomina 1985	93	186	Ham and sausage	2.87	Significantly ↑ risk of CRC
[36] Haenszel et al. 1973	179	357	Sausage and other processed pork	2.3Ϟ 1.77 Ф 2.7҂	Significantly ↑ risk of CRC
[35] Levi et al. 2004	323	1271	Ham salami sausage	2.53 (1.50–4.27)	Significantly ↑ risk of CRC
[38] Bidoli et al. 1992	123¥ 125₸	699	Salami and sausages	1.8¥ 1.9₸	Sig ↑¥ but not ₸
[37] Young and Wolf 1988	152ⱡ 201‡	618	Processed lunch meat	1.85 (1.33–2.58)	Significantly ↑ risk of CRC
[42] De Verdier et al. 1991	559	505	Bacon	1.3 (0.8–1.9)¥ 1.7 (1.1–2.8)₸	Sig ↑ ¥ but not ₸
[39] Navarro et al. 2003	287	566	Cold cuts and sausages	1.64 (1.16–2.32)	Significantly ↑ risk of CRC
[62] Dales et al. 1978	99	280	Nitrite treated meats	1.22	No significant risk of CRC
[43] Macquart-Moulin 1986	399	399	Charcuterie	0.89	No significant risk of CRC
[80] Iscovich et al. 1992	110	220	Delicatessen meat	0.43 (0.21–0.89)	Significantly ↓ risk of CRC
[45] Nowell et al. 2002	157	380	Sausage and bacon	-	Significantly ↑ bacon consumption in cases group

↑ increased; ↓ decreased; ⱡ Proximal colon; ‡ distal colon; CRC colorectal cancer, ¥ colon, ₸rectum, ♂ male; ♀ female; Ϟ Hawaiian; Ф Issei; ҂Nisei. Where the authors did not provide the relative risks for males and females combined, we have provided the gender specific relative risks. Relative risk values reflect those in the highest consumption group vs those in the lowest consumption group. All relative risk values are adjusted, with the exception of Iscovich et al. and Nowell et al. more details of adjustments are provided in Supplementary Tables S1 and S2.

## Supplementary Materials

The following are available online at https://www.mdpi.com/2072-6643/11/11/2673/s1: Table S1: Characteristics of prospective human studies assessing colorectal cancer and processed meat consumption; Table S2: Characteristics of case control human studies assessing colorectal cancer and processed meat consumption.
